# Favourable Changes in C-Peptide, C-Reactive Protein and Lipid Profile, and Improved Quality of Life in Patients with Abnormal Body Mass Index after the Use of Manual Lymphatic Drainage: A Case Series with Three-Month Follow-Up †

**DOI:** 10.3390/medicina58020273

**Published:** 2022-02-11

**Authors:** Klaudia Antoniak, Katarzyna Zorena, Rita Hansdorfer-Korzon, Dagmara Wojtowicz, Marek Koziński

**Affiliations:** 1 Department of Immunobiology and Environment Microbiology, Medical University of Gdańsk, Dębinki 7, 80-211 Gdańsk, Poland; 2 Department of Physical Therapy, Medical University of Gdańsk, Dębinki 7, 80-211 Gdańsk, Poland; rita.hansdorfer-korzon@gumed.edu.pl; 3 Department of Cardiology and Internal Diseases, Institute of Maritime and Tropical Medicine, Faculty of Health Sciences, Medical University of Gdańsk, Powstania Styczniowego 9b, 81-519 Gdynia, Poland; dagmara.wojtowicz@gumed.edu.pl (D.W.); marek.kozinski@gumed.edu.pl (M.K.)

**Keywords:** obesity, overweight, low-grade inflammation, dyslipidaemia, quality of life, manual lymphatic drainage

## Abstract

Aim: to try to assess the effect of manual lymphatic drainage on the biochemical parameters and quality of life of patients with abnormal body mass index. The study included three women, average age 46 years (patient 1 with normal body weight as a control; patient 2: overweight; patient 3 with class 2 obesity). After qualification, physiotherapeutic interview and examination was carried out; the concentrations of glycosylated haemoglobin (HbA1c), C-peptide, high-sensitivity C-reactive protein (hsCRP), lipid profile, and quality of life were also examined. Additionally, in patients with abnormal body mass index, biochemical parameters were monitored for 3 months. Each patient underwent 10 manual lymphatic drainage (MLD) therapy sessions, three times a week for 30 min. In the overweight patient (patient 2), a decrease in the concentration of C-peptide, hsCRP and triglycerides was observed after the series of MLD therapy. An improvement in the quality of life, intestinal motility, and a reduction in the frequency of flatulence were also noted. Moreover, after the therapy, patient 2 reported better sleep and increased vitality. In contrast, in patient 3 (with grade 2 obesity), a decrease in triglyceride levels, but not other biomarkers, was detected after the series of MDL therapy. Additionally, in patient 3, an improvement in the quality of life, an improvement in intestinal peristalsis, and reduction of menstrual pain were observed after MLD therapy. For comparison, in a patient with a normal body weight as a control (patient 1), there were no changes in biochemical parameters or improvement in the quality of life after MLD therapy. Our preliminary research indicates improvement of the concentration C-peptide, lipid profile, a reduction in the inflammation, and improved quality of life in patients with abnormal body mass index after MLD therapy. However, more studies are needed to elucidate the effectiveness of MLD therapy in patients with varying degrees of abnormal body mass index, i.e., from overweight to obesity.

## 1. Introduction

According to the World Health Organization (WHO), obesity is defined as “abnormal or excessive fat accumulation that presents a risk to health” [1]. Currently, it is a rapidly spreading, non-communicable pandemic with a global incidence of 39%. Previous studies have demonstrated the multifactorial aetiology of the condition, with genetic, endocrine, metabolic, environmental, and psychological–behavioural factors playing a significant role [2,3,4]. The consequences of abnormal body weight in relation to height include an increased risk of many diseases [5,6]. The studies conducted so far have shown that the risk of obesity-related diseases depends not only on the degree of obesity, but also on the distribution of the adipose tissue [7,8,9,10]. Abdominal (central) obesity increases the risk of diabetes, hypertension, and atherosclerosis [7]. Gluteal-femoral obesity favours the development of hormone-dependent cancers of the uterus, ovary, breast, and gastrointestinal tract [7,8]. Moreover, depending on the anatomical location, the adipose tissue deposits differ in terms of the metabolic activity. The subcutaneous tissue is less metabolically active than the visceral tissue, which means that not only the size of a particular type of the adipose tissue, but also its distribution is important for health [8,9]. Visceral adipose tissue stimulates a significant influx of inflammatory cells and acute-phase proteins, such as e.g., C-reactive protein (CRP), leading to low-grade inflammation and, over the years, can lead to chronic inflammation and chronic vascular complications [10,11,12]. Moreover, in patients with abnormal body mass index, there is dysregulation of lipid metabolism, including an increase in triglycerides, total cholesterol, and LDL cholesterol while lowering HDL cholesterol. There may also be disturbed carbohydrate metabolism [13,14,15,16].

**Figure 1:**
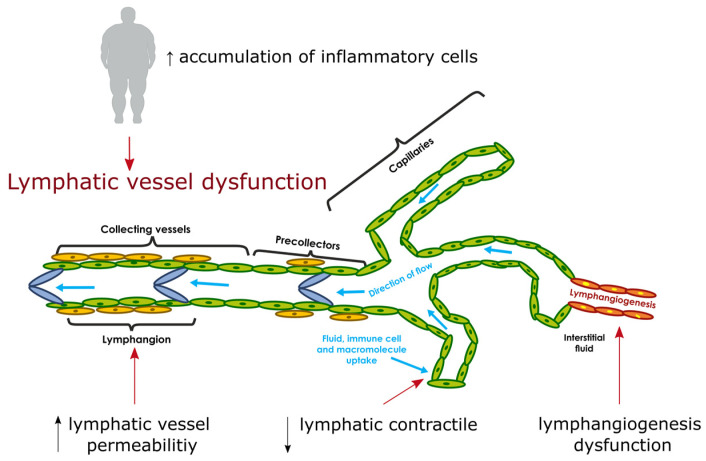
Dysfunction of the lymphatic system in obesity. Modified Figure 1 [24]. Abbreviations: ↑—increase, ↓—decrease.

In recent years, several studies have been conducted showing that obesity may cause pathological changes in the lymphatic system, which may impair its function, and conversely, dysregulation of the lymphatic system may lead to obesity [16,17,18]. The disturbed lymphatic flow, observed in people with abnormal body weight may lead to changes in the composition of interstitial fluid, and thus affect the adipocyte microenvironment [16]. Moreover, as a result of the disturbed function of the lymphatic system, water retention was observed in the extracellular space, along with the accumulation of a fluid rich in protein containing lipids, and this leads to an increase in colloid osmotic pressure [19,20]. Lymphostasis may cause the accumulation of inflammatory cells as well as cytokines, chemokines, and growth factors at the site of swelling, involving the lymphatic system in the immune response [21]. Additionally, in the case of obesity, decreased lymphatic endothelial cell (LEC) proliferation, increased lymphatic permeability, decreased lymphatic vessel density, and reduced lymphatic contractility are observed as shown in Figure 1 [17,22,23].

In the twenty-first century, patients with excessive body weight should be covered by interdisciplinary care tailored to individual needs. These ought to include, first of all, a properly balanced diet, appropriately selected physical activity and/or pharmacological as well as non-pharmacological treatment, including physiotherapy [25,26,27,28]. In light of the above reports, we hypothesized that the use of therapy, including manual lymphatic drainage (MDL), may improve the dysfunction of the lymphatic system in people with abnormal body mass index. Manual lymphatic drainage is founded on the stimulation ofthe lymphatic system by increasing lymph circulation, expediting the removal of harmful metabolites from body tissues and enhancing body fluid dynamics [24,29]. So far, MLD has been applied in the treatment of lymphoedema and lipoedema [30,31]. Additionally, there have been reports suggesting the positive influence of MLD on the treatment of women with infertility [32], as well as people with acne rosacea accompanied by chronic lymphoedema of the face [33]. Other studies show the effectiveness of MLD in supporting the treatment of burns [34], as well as reducing the alpha brain wave activity in patients with mental stress [35]. In turn, Arngrim et al. investigated subcutaneous adipose tissue lymphatic drainage of macromolecules in lean and obese subjects and evaluated whether adipose tissue lymphatic drainage may change in parallel with adipose tissue blood flow [36]. It was shown that a significant increase in lymphatic drainage occurred after glucose loadingin the lean subjects. In the obese subjects, lymphatic drainage remained constant throughout the study and was significantly lower compared to the lean subjects. The authors suggest that the obtained results indicate a reduced ability to remove macromolecules from the interstitial space through the lymphatic system in obese subjects. This may cause a high local production of pro-inflammatory cytokines and, as a consequence, the development of obesity-related inflammation in hypertrophic adipose tissue [36]. Therefore, it is important to search h for therapeutic interventions aimed at supporting the treatment of obesity, including the reduction of comorbid chronic inflammation. The aim of our study was to try to assess the effect of manual lymphatic drainage on the biochemical parameters and quality of life of patients with abnormal body mass index.

## 2. Materials and Methods

The study was approved by the Ethics Committee of the Medical University of Gdańsk (approval no. NKBBN/692/2019–2020; approval date: 30 January 2020) and the investigation was carried out in accordance with the principles of the Declaration of Helsinki as revised in 2013. Three women were included in the study, the average age of the subjects was 46 years. Patient number 1 was 49 years old, professionally active (blue-collar worker). The patient’s body weight was normal (BMI = 22 kg/m^2^), she was highly physically active and had positive family history of diabetes. The WHR (Waist–Hip Ratio) was below the limit of abdominal obesity and amounted to 0.73. Her blood pressure was 120/66 mmHg. The medical history included uterine myomas; at the time of the study the patient was being diagnosed for severe menstrual pain. She declared no chronic diseases and did not take any medications. Patient number 2 was a woman at the age of 59, professionally active (white-collar worker), overweight (BMI = 27 kg/m^2^), abdominal obesity (WHR 0.84), low physical activity. The mean blood pressure was 122/85 mmHg. The patient was under the constant care of a gastroenterological outpatient clinic; she was regularly taking dexlansoprazole (Dexilant, Takeda Pharma, Japan) at a dose of 30 mg 3 times a week.

Patient number 3, aged 30, professionally active (white-collar worker). The patient hadclass 2 obesity (BMI = 35 kg/m^2^), abdominal obesity (WHR index 0.9), moderate physical activity. The mean blood pressure was 120/77 mmHg. The medical history revealed heart rhythm problems, insulin resistance and polycystic ovary syndrome. The family history was positive for hypertension, dyslipidaemia, and ischemic heart disease. The patient did not take any medications. The characteristics of the subjects are presented in Table 1.

**Figure 2:**
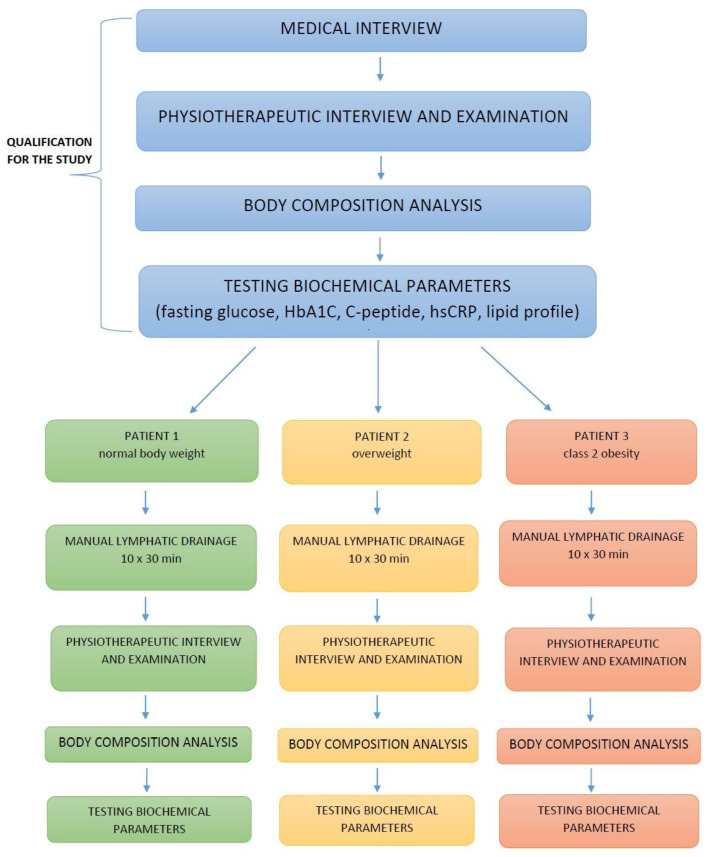
Participant flow diagram.

After qualification, each patient underwent a biochemical test performed on the first visit. The fasting glucose level was assessed using the hexokinase method and spectrophotometry (Cobas 8000 analyser, Roche, Switzerland). The level of glycosylatedhaemoglobin (HbA1c) was determined by immunoturbidimetry using the Cobas 8000 analyser (Roche, Basel, Switzerland); the level C-peptide was measured by the CMIA method. The concentration of high-sensitivity C-reactive protein (hsCRP) was assessed by immunoturbidimetry (Cobas 8000 analyser, Roche, Switzerland). In addition, the level of total cholesterol and triglycerides was determined in the serum by colorimetry using the Cobas 8000 analyser, Roche, Switzerland; the levels of LDL and HDL cholesterol were determined by direct colorimetry (Cobas 8000, Roche, Switzerland). The patients were examined according to the scheme presented in Figure 2. During the first and last physiotherapeutic visit, the patients were interviewed individually (Figure 2).

The questions involved self-assessment of health and lifestyle. The Likert scale (1–7) was applied to evaluate the quality of life: “1—definitely poor”, “2—poor”, “3—fairly satisfactory”, “4—satisfactory”, “5—good”, “6—very good”, “7—excellent” [37]. For each patient, anthropometric measurements were taken using the same scales. Height measurements were performed with an accuracy of 0.5 cm and body weight measurements with an accuracy of 0.1 kg. The body mass index (BMI) was calculated according to the formula: BMI = body weight (kg)/height (m)^2^. Waist circumference was measured in a standing position (halfway between the costal arches and the upper edge of the iliac crest); hip circumference (at the height of the trochanter major) by means of an inelastic measure (with an accuracy of 0.5 cm). Then, the waist-to-hip ratio (WHR) was calculated for each patient according to the formula: WHR=waist circumference (cm)/hip circumference (cm). The degree and type of obesity was determined on the basis of the BMI index and the WHR index according to the guidelines of the Polish Diabetological Society [38]. In addition, each patient was informed not to change theirlifestyle and eating habits during the MLD therapy and until the next blood test wasdone. The biochemical parameters in patient 1 was performed at 2 points:at point 0′(before therapy) and at point 1′ one month after therapy). In the case of patients 2 and 3, the biochemical parameters were performed at 4 points:at point 0′(before therapy), at point 1′ (one month after therapy), at point 2′(two months after therapy) and at point 3′ (three months after therapy).

**Figure 3:**
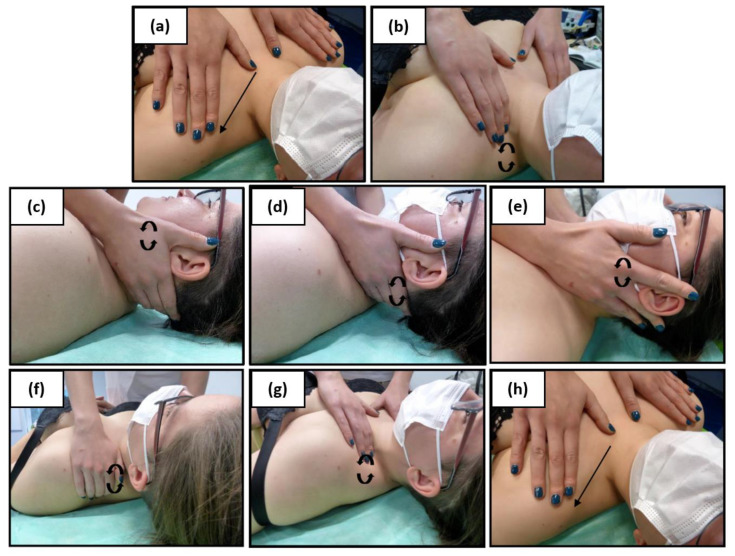
Pretreatment: Manual lymphatic drainage of the neck; effleurage: superficial strokes from the sternum to the acromion (**a**); stationary circles in the supraclavicular fossa on the lower deep cervical lymph nodes (**b**); stationary circles from the mandibular angle, over the upper and lower deep cervical lymph nodes (**c**); stationary circle along the nuchal line, occipital lymph nodes (**d**); stationary circles in front of and be-hind the ear (**e**); stationary circles from the acromion posteriorly to the spine of the scapula to the supraclavicular fossa (**f**); stationary circles in the supraclavicular fossa (**g**); superficial strokes from the sternum to the acromion (**h**).

**Figure 4:**
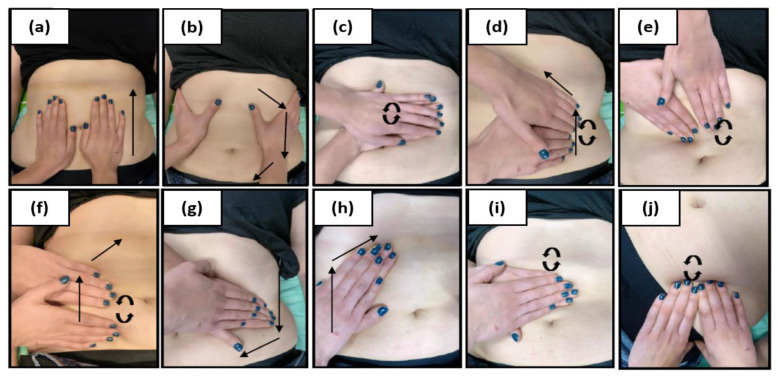
Manual lymphatic drainage of the abdomen; effleurage: during inhalation, rotary technique from the pubic bone to the sternum (**a**); during exhalation, rotary technique from the costal arch and the iliac crest back toward the pubic bone (**b**); circles over the cisterna chili and the course of the large intestine (**c**); stationary circles over the descending (**d**), ascending (**e**) and transverse colon(**f**) withthe pressure applied in the direction of the cisterna chili; stroke of seven: circular strokes over the descending colon from the spleen area to the bladder (**g**); circular strokes over the ascending colon to the liver area (**h**); stationary circles over the naval and return to the spleen area (**i**);therapy of the iliac lymph nodes (**j**).

**Figure 5:**
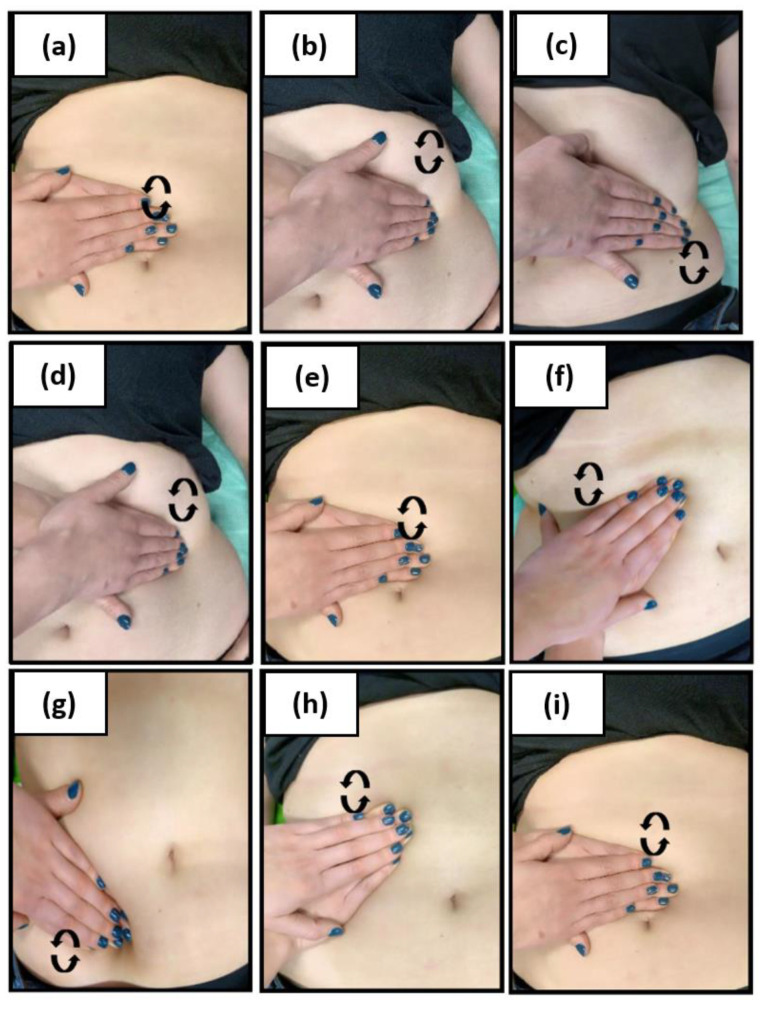
Abdominal deep drainage: on the naval (**a**); parallel to the left costal arch (**b**); parallel to the left inguinal ligament (**c**); parallel to the left costal arch (**d**); on the naval (**e**); parallel to the right costal arch (**f**); parallel to the right inguinal ligament (**g**); parallel to the right costal arch (**h**); on the naval (**i**); conclusion effleurage with breathing.

During the first and subsequent physiotherapeutic visits, blood pressure was taken after 15 min of rest using an arm sphygmomanometer. After that, each subject underwent 10 MLD therapyaccording to Földi [29], which covered the abdominal cavity, groin area and the neck area (Figure 3, Figure 4 and Figure 5). In all patients, one-time therapy lasted 30 min and was carried out three times a week. MLD was performed in the supine position with the lower limbs slightly abducted and flexed at the knee joints (in order to relax the abdominal wall); the upper limbs were positioned along the body.

One month after a series of 10 MLD therapies, the subjects underwent another biochemical test, in which the same parameters were examined, before and after the MLD therapy. Because there were no changes in the level of biochemical parameters in patient 1, the biochemical examination was performed only once after the MDL therapy. The test results are presented in Table 2. The biochemical examination was repeated in patient 2 and patient 3 two months and three months after the MLD therapy. The results are presented in Table 3 and Table 4, respectively.

## 3. Results

Patient 1: The physical examination revealed normal body weight (BMI = 22 kg/m^2^), no abdominal obesity (WHR = 0.73), and mean blood pressure 120/66 mm Hg. During the therapy, patient 1 reported somnolence, which subsided after MLD. After the therapy, the patient observed relief in menstrual pain and decreased intensity of PMS symptoms (reduced breast tenderness, no lower abdominal pain or sleep disturbance); these effects lasted for three consecutive menstrual cycles. The quality of life scored according to the Likert scale was 6 points before vs. 6 points after the MLD therapy. In patient 1, before and after the MLD therapy, the parameters of inflammation and carbohydrate metabolism, as well as the levels of triglycerides (TG) and high-density-lipoprotein cholesterol (HDL-C) were within the reference values; the concentrations of total cholesterol and low-density-lipoprotein cholesterol (LDL-C) exceeded the reference values. In patient 1, the HbA1C level was 5.6% before vs. 5.6% after the MLD therapy, the fasting glucose was 97 vs. 94 mg/dL respectively, the concentration of C-peptide was 1.16 vs. 1.21 ng/mL, while the hsCRP level was <1 vs. 2 mg/L. As for lipid metabolism, the concentration of total cholesterol was 213 vs. 214 mg/dL, HDL-C 67 vs. 66 mg/dL, LDL-C 135 vs. 134 mg/dL, and TG 51 vs. 67 mg/dL. The results are presented in Table 2.

Patient 2: The anthropometric measurements showed that patient 2 was overweight (BMI = 27 kg/m^2^) and had abdominal obesity (WHR index = 0.84). The medical history revealed frequent constipation, flatulence, gastroesophageal reflux, oesophageal erosion, and headaches. Her blood pressure was 122/85 mm Hg. During the MLD therapy, the overweight patient reported increasing headaches and higher heart rate; the symptoms resolved after the MLD therapy. An improvement in intestinal motility and a reduction in the frequency of flatulence were observed after a series of MLD therapy. Moreover, after the therapy, the patient reported better sleep and increased vitality. During a follow-up conducted three months after the MLD therapy, the patient declared that an improvement in all the above-mentioned components maintained. In the Likert scale, the quality of life was scored at 3 points before vs. 5 points after the MLD therapy.As for biochemical parameters, before and after MLD therapy, the patient showed higher levels of total cholesterol and LDL-C compared to the reference values. On the other hand, a decrease in the level of HDL-C, TG, hsCRP and C-peptide was detected after the MLD therapy. The results for patient 2 are presented in Table 3.

Patient 3: Class 2 obesity (BMI = 35 kg/m^2^), (WHR = 0.9). The blood pressure was 120/77 mm Hg. The medical history revealed constipation and menstrual pain. During the MLD therapy, the patient reported increased sleepiness and greater appetite. After the therapy, an improvement in intestinal peristalsis and reduction of menstrual pain were observed. The reduction in menstrual pain lasted for four consecutive menstrual cycles. On the other hand, the digestive system ailments returned after the MLD therapy. The quality of life scored on the Likert scale was 3 points before vs. 4 points after the therapy. In the patient with class 2 obesity, the levels of total cholesterol and LDL were higher after the MLD. However, a significantly lower concentration of triglycerides was detected before the therapy (131 vs. 67 mg/dL). Moreover, after MDL therapy, a decrease as well as an increase in the level of C-peptide and hsCRP protein was observed. In an overweight patient, before MLD, the C-peptide level was 2.4 and 1.9 ng/mL one month after therapy; after two months, there was an increase in the C-peptide level of 2.03 ng/mL, and after three months, the level of C-peptide was 2.23 ng/mL (Table 4). Before manual therapy of the abdominal cavity, the hsCRP level was 1.3 vs. 1.9 mg/mLone month after therapy,2.1 mg/mLtwo months after therapy, and 1.6 mg/mLafter three months. The results for patient 3 are shown in Table 4.

## 4. Discussion

Three patients were examined, including one with a normal body weight as a control and two patients with an abnormal body weight, i.e.,an overweight patient (patient 2) and a patient with class 2 obesity (patient 3). In all of the subjects, the levels of total cholesterol and LDL were above the reference values before and after the MLD therapy. However, a decrease in TG levels was observed in an overweight patient and a patient with class 2 obesity. We suggest that one of the reasons for lowering the TG level in the overweight patient and in the patient with class 2 obesity may be the use of manual lymphatic drainage. So far, no study of the effect of MLD therapy on the lipid profile in patients with abnormal body weight has been described in the available scientific databases.Therefore, our results apply to other forms of manual therapy, such as mechanical massage and manual acupuncture [39,40]. The decrease in the level of triglycerides in our patients with abnormal body weight is consistent with the results obtained by Marques et al., who demonstrated a reduction in the synthesis of triglycerides after mechanical massage in female gluteofemoral adipose tissue [39]. In anotherstudy, the authors demonstrated the effect of manual acupuncture on the blood-lipid, hsCRP and adiponectin levels in hyperlipidaemia rats. The researchers showed that the manual acupuncture therapy performed in hyperlipidaemia rats led to up regulating serum high density lipoprotein cholesterol and adiponectin levels and also a lower concentration of lipid profile including triglycerides and hsCRP level [40]. Interestingly, in our overweight patient (patient 2), a decrease in the serum level of hsCRP was also observed after a series of the manual therapy. The decrease in hsCRP level observed in an overweight patient may indicate a reduction in inflammation after the use of a series of manual lymph drainage therapy. In contrast, in patient 1, with a normal body weight as a control, no changes in the level of hsCRP were observed. On the other hand, in patient 3, with class 2 obesity, the so-called “CRP levels swing” was observed after the MLD therapy. Fluctuating levels of hsCRP in the blood may be due to the fact that the patient with class 2 obesity suffered from insulin resistance, cardiac arrhythmias and polycystic ovary syndrome (PCOS). The condition increases the risk of ischemic heart disease and type 2 diabetes. Moreover, some authors have demonstrated mild chronic systemic inflammation as an important risk factor for PCOS [41,42]. The markers of inflammation, such as hsCRP, tumour necrosis factor α (TNFα), interleukin-1α (IL-1α), interleukin-1b (IL-1b), interleukin-6 (IL-6) and interleukin-18 (IL-18) have been detected in PCOS patients [41].

To the best of our knowledge, in the available scientific databases, the effects of MLD therapy on hsCRP levels have not been assessed in any of the studies; however, several studies have shown that MLD therapy affects other parameters of inflammation [43,44,45]. For instance, in 2011, Huggenberger et al. showed that the MLD therapy reduces inflammation by lowering the release of inflammatory factors, such as prostaglandins, bradykinin and histamine. It also decreases the production of inflammatory cells, such as neutrophils, macrophages, and lymphocytes [43]. Similar conclusions were presented by Schwartz et al., who indicated the potential benefits of the MLD therapy for improving the lymphatic function and reducing inflammation in autoimmune diseases [44]. Apart from that, our results may be comparable to those of the study conducted by Shahrjejdi [45].The authorshowed that classicmassage for eight weeks is an efficient method forreducing the C-reactive protein in patients with obesity [45].

Moreover, we analysed the effect of MLD therapy on the concentration of C-peptide in patients with abnormal body weight. In our study, before the MLD therapy, all the patients showed levels of C-peptide within the reference standards, with the lowest value being reported in the patient with normal body weight. In control patient 1, with normal body weight, no changes in the level of C-peptide were observed after series of MLD therapy. On the other hand, in patient 3, with class 2 obesity, the so-called “C-peptide levels swing” was observed after the MLD therapy.

Interestingly, however, after a series of MLD therapy, an overweight patient showed a constant decrease in the serum level of C-peptide. We believe that a decrease in the level of C-peptide may be an effect after use of MLD, which is desired in the prevention of insulin resistance and/or type 2 diabetes [46].

To the best of our knowledge, this is the first study to evaluate the effect of MLD therapy on serum C-peptide levels in patients with abnormal body weight. In the study by Andrade et al., which lasted 30 years, a positive correlation was shown between C-peptide concentration and abdominal obesity, BMI, glucose level, high-sensitivity C-reactive protein and triglycerides, while the correlation was negative with HDL cholesterol level [46].

In addition, apart from the obvious impact on physical functioning, obesity is related to a significant reduction in the quality of life [47,48,49]. Patients with obesity may experience lack of social acceptance, which results in social exclusion or social discrimination [50]. The studies indicated a positive correlation between obesity and psychological problems and increased risk of psychiatric disease [49] and depression [48].

An important result of our research is that patients 2 and 3 showed a better quality of life based on the Likert scale and an increased vagotonic effect relaxation, as well as better intestinal peristalsis and sleep quality after MDL therapy. In the overweight patient, this effect lasted for three months, while in the patient with class 2 obesity it lasted for two months after the therapy. In patient 2, with overweight, an improvement in intestinal motility and a reduction in the frequency of flatulence were observed after a series of MLD therapy. Moreover, after the therapy, the patient reported better sleep and increased vitality. During a follow-up conducted three months after the MLD therapy, the patient declared that an improvement in all the above-mentioned components maintained. In the Likert scale, the quality of life was scored “quite satisfactory” before MLD vs. “good” after MDL therapy (3 points before vs. 5 points after the MLD therapy). In the patient with class 2 obesity after MLD therapy, an improvement in the quality of life was shown according to Likert scale from “fairly satisfactory” to “satisfactory” (3 points before MLD therapy vs. 4 points after MLD therapy). Furthermore, the patient with class 2 obesity reported an improvement in intestinal peristalsis and a decrease in menstrual pain, lasting from three to four menstrual cycles after the MLD therapy.

Previous studies, including the research conducted by our team, prove the effectiveness of physiotherapeutic methods in the treatment of menstrual ailments [51]. In turn, other researchers have proved the effectiveness of including the lymphatic techniques in supporting the treatment of infertility [32]. A positive effect of manual therapy (massage, visceral therapy) on intestinal peristalsis and reduction of constipation in the elderly hasalso been demonstrated [52,53].

The preliminary results of our study are of clinical importance because we have shown that after MDL therapy there may be a reduction in the level of selected biochemical parameters. In addition, improvement in intestinal peristalsis and reduction of menstrual pain may be a measurable indicator of improvement in the quality of life in patients with abnormal body mass index.

## 5. Study Limitations

The present study has some limitations that we would like to address. First, our study did not include a patient without manual lymphatic drainage and therefore we cannot rule out a placebo effect. Second, the research presented was based on a small group of women, without any men included. Importantly, future studies should be expanded to include the examinations of differences between genders, ages, or longer therapy durations. We also acknowledge that the case series design limits us from drawing definitive conclusions about the benefit of manual lymphatic drainage for all individuals with abnormal body mass index. Further cohort studies are being conducted to verify our findings and to garner a deeper understanding MDL therapy in patients with abnormal body mass index.

## 6. Conclusions

The preliminary studies demonstrated that MLD may be one of the therapies supporting the function of the lymphatic system in patients with abnormal body mass index and have a positive effect on biochemical parameters. Additionally, the therapy is a well-tolerated and relatively inexpensive method of improving the function of the lymphatic function used to reduce mild chronic inflammation associated with obesity. To our knowledge, these are the first data supporting the hypothesis that the patients with abnormal body mass index, who are treated with MLD, have a measurable improvement in biochemical parameters, and better quality of life. More research and multivariate analysis will be required to elucidate a definitive mechanism of the MLD therapy effectiveness.

## Figures and Tables

**Table 1:** Characteristics of the subjects with normal body weight, overweight, and class 2 obesity.

Parameter	Patient 1	Patient 2	Patient 3
Age [years]	49	59	30
Education	secondary	higher	secondary
Professional work	physical work	mental work	mental work
BMI	22	27	35
	normal	overweight	Class 2 obesity
WHR [cm]	0.73 (normal)	0.84 (abdominal obesity)	0.9 (abdominal obesity)
Blood pressure [mm Hg]	120/66	122/85	120/77
Comorbidities	no comorbidities	Gastro-oesophageal reflux	polycystic ovary syndrome, insulin resistance
Constipation	+	+	−
Flatulence	−	+	+
Dysmenorrhoea	+	−	+
Headaches	−	+	−
Physical exercise	5 times a week	never	1–2 times a week
Consuming 1.5 litres of water daily	yes	no	yes
Consuming sweets daily	no	yes	yes

**Table 2:** Biochemical parameters before and after the MLD therapy in a patient with normal body weight.

Parameter	0′	1′
Age [years]	49	49
Blood pressure [mmHg]	120/66	120/66
HbA1C [%]	5.6	5.6
Fasting glucose [mg/dL]	97	94
C-peptide [ng/mL]	1.16	1.21
hsCRP [mg/L]	< 1	2
Total cholesterol [mg/dL]	213	214
HDL-C [mg/dL]	67	66
LDL-C [mg/dL]	135	134
TG [mg/dL]	51	67

Abbreviations: 0′, before therapy; 1′, one month after MLD therapy; HbA1C, glycosylated haemoglobin); hsCRP, high-sensitivity C-reactive protein; HDL-C, high-density-lipoprotein cholesterol, LDL-C, low-density-lipoprotein cholesterol; TG, triglycerides.

**Table 3:** The results of biochemical parameters before and after the MLD therapy in an overweight patient.

Parameter	0′	1′	2′	3′
Age [years]	59	59	59	59
Blood pressure [mm Hg]	122/85	122/85	122/85	122/85
HbA1C [%]	5.5	5.6	5.7	5.5
Fasting glucose [mg/dL]	91	95	91	88
C-peptide [ng/mL]	2.95	2.23	2.19	1.94
hsCRP [mg/L]	3.6l	3	1.3	<1
Total cholesterol [mg/dL]	253	239	259	268
HDL-C [mg/dL]	51	47	53	50
LDL-C [mg/dL]	177	169	180	199
TG [mg/dL]	125	109	130	97

Abbreviations: 0′, before therapy; 1′, one month after MLD therapy; 2′, two months after MLD therapy; 3′, three months after therapy; HbA1C, glycosylated haemoglobin; hsCRP, high-sensitivity C-reactive protein; HDL-C, high-density-lipoprotein cholesterol; LDL-C, low-density-lipoprotein cholesterol; TG, triglycerides.

**Table 4:** The values of biochemical parameters before and after the MLD therapy in a patient with class 2 obesity.

Parameter	0′	1′	2′	3′
Age [years]	30	30	30	30
Blood pressure [mmHg]	120/77	120/77	120/77	120/77
HbA1C [%]	5.6	5.6	5.6	5.7
Fasting glucose [mg/dL]	105	105	101	106
C-peptide [ng/mL]	2.4	1.9	2.03	2.23
hsCRP [mg/L]	1.3	1.9	2.1	1.6
Total cholesterol [mg/dL]	194	211	225	223
HDL-C [mg/dL]	57	55	59	65
LDL-C [mg/dL]	111	144	150	145
TG [mg/dL]	131	61	79	67

Abbreviations: 0′, before therapy; 1′, one month after MLD therapy; 2′, two months after MLD therapy; 3′, three months after MLD therapy; HbA1C, glycosylated haemoglobin; hsCRP, high-sensitivity C-reactive protein; HDL-C, high-density-lipoprotein cholesterol; LDL-C, low-density-lipoprotein cholesterol; TG, triglycerides.

## Data Availability

The data presented in this study are available on request from the corresponding author: klaudia.antoniak@gumed.edu.pl.
